# The Grief and Bereavement Experiences of Informal Caregivers: A Scoping Review of the North American Literature

**DOI:** 10.1177/08258597211052269

**Published:** 2021-12-03

**Authors:** Neerjah Skantharajah, Carol Barrie, Sharon Baxter, M. Carolina Borja, Anica Butters, Deborah Dudgeon, Ayeshah Haque, Iqra Mahmood, Mehrnoush Mirhosseini, Raza M. Mirza, Ankita Ankita, Carly Thrower, Christina Vadeboncoeur, Andrew Wan, Christopher A. Klinger

**Affiliations:** 1 University of Toronto, Toronto, Ontario, Canada; 2 Canadian Frailty Network (CFN), Kingston, Ontario, Canada; 3 Quality End-of-Life Care Coalition of Canada (QELCCC), Ottawa, Ontario, Canada; 4 Canadian Hospice Palliative Care Association (CHPCA), Ottawa, Ontario, Canada; 5 Canadian Partnership Against Cancer (CPAC), Toronto, Ontario, Canada; 6 Queen's University, Kingston, Ontario, Canada; 7 College of Family Physicians of Canada (CFPC), Mississauga, Ontario, Canada; 8 University of Alberta, Edmonton, Alberta, Canada; 9 National Initiative for the Care of the Elderly (NICE), Toronto, Ontario, Canada; 10 Canadian Network of Palliative Care for Children (CNPCC), Ottawa, Ontario, Canada; 11 University of Ottawa, Ottawa, Ontario, Canada; 12 Pallium Canada, Ottawa, Ontario, Canada; 13 McMaster University, Hamilton, Ontario, Canada

**Keywords:** anticipatory grief, bereavement, complicated grief, hospice and palliative care, informal caregivers, older adults, scoping review

## Abstract

**Background:** Informal caregivers are a significant part of the hospice and palliative care landscape as members of the interdisciplinary care team. Despite this, little is known about the impact this responsibility has on informal caregivers’ experiences of grief and bereavement. **Objective:** To address this, a scoping review of the literature was conducted to explore the current state of knowledge toward grief and bereavement of informal caregivers of adult/geriatric patients in the hospice and palliative/end-of-life care realm within North America. **Methods:** Using Arksey and O’Malley's 5-step framework, key electronic health care and social sciences databases (eg, CINAHL, MEDLINE, ProQuest Sociological Abstracts, PsycINFO) alongside gray literature sources were searched and screened against inclusion and exclusion criteria. A thematic content analysis was used to identify key themes. **Results:** 29 articles met the final inclusion criteria with 3 central themes emerging: (1) mediators of grief, (2) grief experiences, and (3) types of grief. **Discussion:** Informal caregivers encounter unique grief and bereavement experiences: The range of psychosocial outcomes, both negative and positive, can be affected by various mediators such as caregiver burden, demographics, disease type of the patient being cared for, etc. Bereavement interventions must be designed with the mediators of grief in mind. **Conclusions:** Understanding the nuances of informal caregivers’ experiences with grief and bereavement will inform and advance practice, policy, and research. Practitioners/clinicians should be further educated on how to properly acknowledge the complexity of grief and bereavement for informal caregivers, specifically paying attention to mediators. Further research needs to consider the role of culture.

## Introduction

### Informal Caregiving and Grief and Bereavement in Hospice and Palliative Care

In the fields of hospice and palliative care, family members often hold the important position of informal caregiver (IC).^1^ Defined as any family member or friend who provides physical and/or psychosocial care to a person receiving care, ICs occupy a unique role in the landscape of North American hospice and palliative care.^2^ Recently, the Canadian Hospice Palliative Care Association (CHPCA) estimated that over 3 million Canadians (8%) provided informal hospice palliative care,^2^ while in the United States a national survey revealed that around 10.5 million adults (5%) provided informal care.^3^

Informal caregivers’ experiences with grief and bereavement are distinctive. Although grief and bereavement are often used interchangeably, research suggests that the former be thought of as a process while the latter be considered a period of time during which grief is experienced.^4,5^ Mourning is another term that is closely related to grief and bereavement, but can be uniquely defined as the manifestation of grief though social or cultural practices such as funerals, visitations, and other customs.^5^ This paper focuses on the grief experiences of informal caregivers during the bereavement period, herein referred to as grief and bereavement experiences.

Furthermore, grief is a nuanced process often associated with symptoms such as sadness, decreased appetite, and an increased risk of psychiatric disorders like major depressive disorder.^6–8^ There are also different types of grief, including but not limited to, anticipatory and complicated grief.^9–11^ Anticipatory grief is experienced before loss happens and is characterized by the anticipation of what is about to be lost.^9,10^ Complicated grief occurs when the period of grief is prolonged and begins to impair an individual's ability to function.^11^

Despite evidence that ICs are susceptible to higher levels of depression, due to the physical and mental burdens associated with caregiving, there remains a limited understanding of how this plays into the larger landscape of their grief and bereavement experiences.^12,13^

### Outlook on Grief and Bereavement

With aging North American populations, there is a growing number of older individuals (generally defined as age 65 + ) with conditions requiring hospice and palliative care, but not enough resources to address all of their needs.^14–16^ Thus, the default burden has been placed on ICs—with little understanding of the effects this may have on their grief and bereavement experiences. Further, the coronavirus-2019 (COVID-19) pandemic has caused high mortality among older age groups, especially those living in long-term care facilities.^17^ This increase in deaths, and the altered circumstances of dying, might further impact the grief and bereavement of ICs who are unable to visit/in isolation due to restrictions put in place in response to the pandemic.^18^ As such, there is a pressing need to understand and address the experience and impact of grief and bereavement on ICs.

The aim of this scoping review was to explore the current state of knowledge toward grief and bereavement of ICs of adult/geriatric patients in the hospice and palliative/end-of-life care realm within North America.

## Methods

This scoping review was conducted between March and November 2020 in accordance with Arksey and O’Malley's 5 stage framework:^19^ (1) identifying the research question, (2) identifying relevant studies, (3) study selection, (4) charting the data, and (5) collating, summarizing, and reporting the results.

### Identifying the Research Question

This review was guided by the research question: “*How do grief and/or bereavement experiences manifest and what are their effects on ICs of adult/geriatric patients in any hospice and palliative/end-of-life care setting in North America?*” A Population, Intervention, Context, Outcome (PICO) format guided the literature search.^20^ The population of interest was ICs of adult/geriatric patients, and the intervention was hospice and palliative, and/or end-of-life care. The context was within North America and outcomes of interest were grief and bereavement of ICs.

### Identifying Relevant Studies

Advanced Google/hand searches and electronic databases in the health and social sciences (AgeLine, CINAHL, Cochrane Library, EMBASE, JSTOR, MEDLINE, Project Muse, ProQuest Sociological Abstracts, PsycINFO, and Scopus) within the University of Toronto library system were used to conduct searches for articles to be included in this scoping review. Keywords and Medical Subject Headings (MeSH) including “bereavement”, “end-of-life”, “family caregivers”, “grief”, “hospice”, “informal caregiving”, “palliative care”, and “psychological distress” were used in various combinations with Boolean operators (eg, AND, OR, NOT) when searching for peer-reviewed scholarly articles and gray literature sources relevant to grief and bereavement in ICs (see Supplemental Material).

### Study Selection

Six independent reviewers (A.A., A.B., A.H., C.M.B., C.T., N.S.) initially screened articles for the review. The eligibility criteria were: (1) written in English, (2) research conducted in North America or stems from a North American context, and (3) explores grief and bereavement of ICs in an adult/geriatric hospice and palliative care setting. Exclusion criteria included articles that explored pediatric, neonatal, or perinatal hospice and palliative care. Study design/type of article was not part of the inclusion criteria since the goal of this review—in line with Arksey and O’Malley's framework^19^— was to capture the full breadth of research available for this topic. The initial selection included articles from January 2000 to May 2020. The cutoff year for the search (2000) was determined after a straw poll of major databases.

### Charting the Data

The full-text review of articles was conducted by 4 independent reviewers (A.W., C.M.B., C.T., I.M.). The 2 reviewers were assigned per article. All articles that had at least 1 reviewer's approval were evaluated by the entire team for final selection and consensus reached. The selected articles were then inputted into a data extraction table (Supplemental Material) which was completed by 4 independent reviewers (A.B., C.M.B., C.T., N.S.). The data extraction table identified article title, author, country of publication, uniform resource locator, publication year, journal, purpose, sample, and setting of care, study design, outcome measures, findings, and practice/policy/research implications. Following data charting, major patterns and themes found within the data were identified utilizing a thematic content analysis approach.^21^

### Collating, Summarizing, and Reporting the Results

The team discussed these emerging major patterns, themes, and overall findings in detail and evaluated their contributions to the overall scoping review purpose and research questions.

Resulting consensus themes/subthemes were cross-referenced and reoccurring ones containing a high volume of quality material were included (see Supplemental Material).

## Results

**Figure 1.**
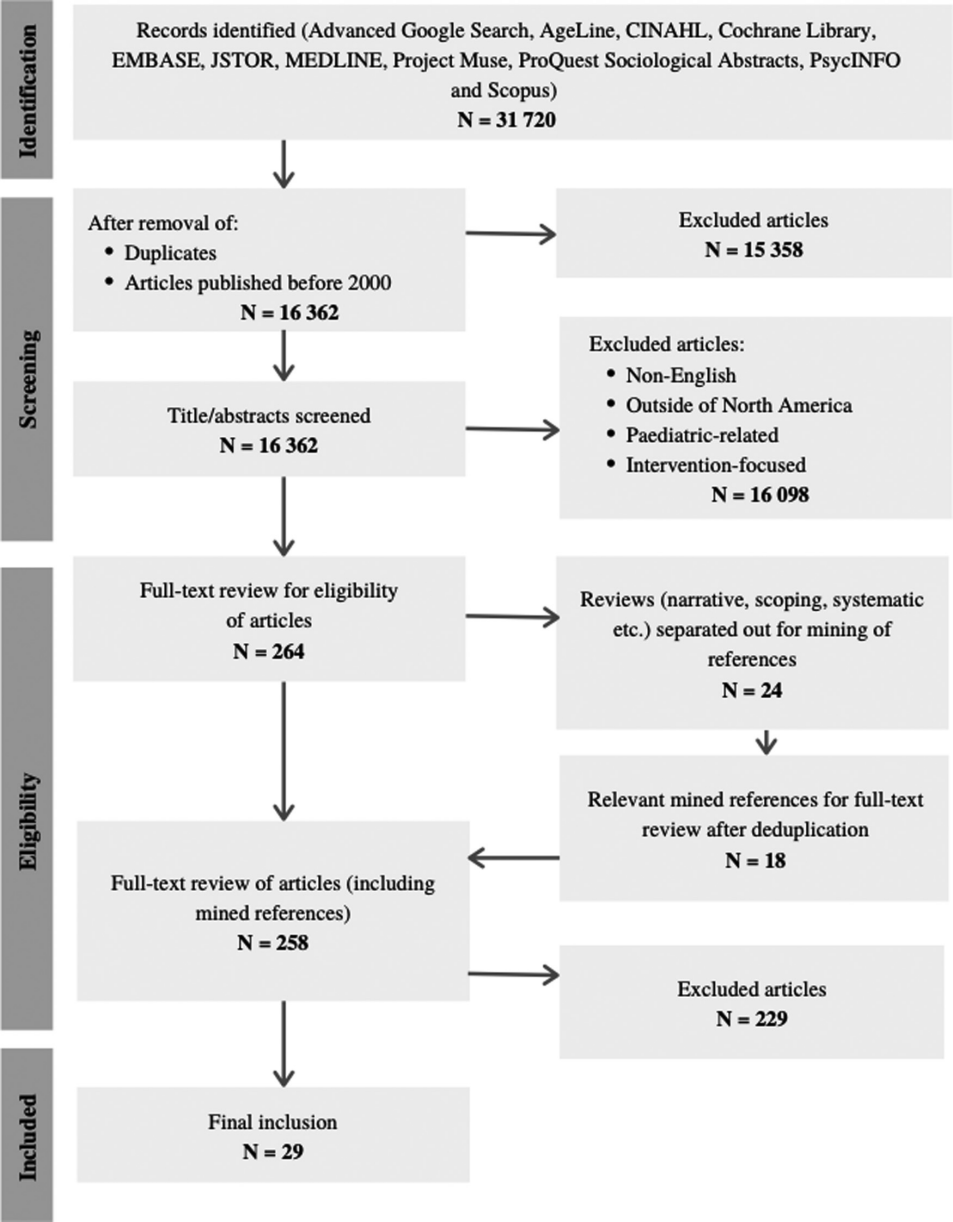
Preferred reporting items for systematic reviews and meta-analyses flow chart.

**Figure 2.**
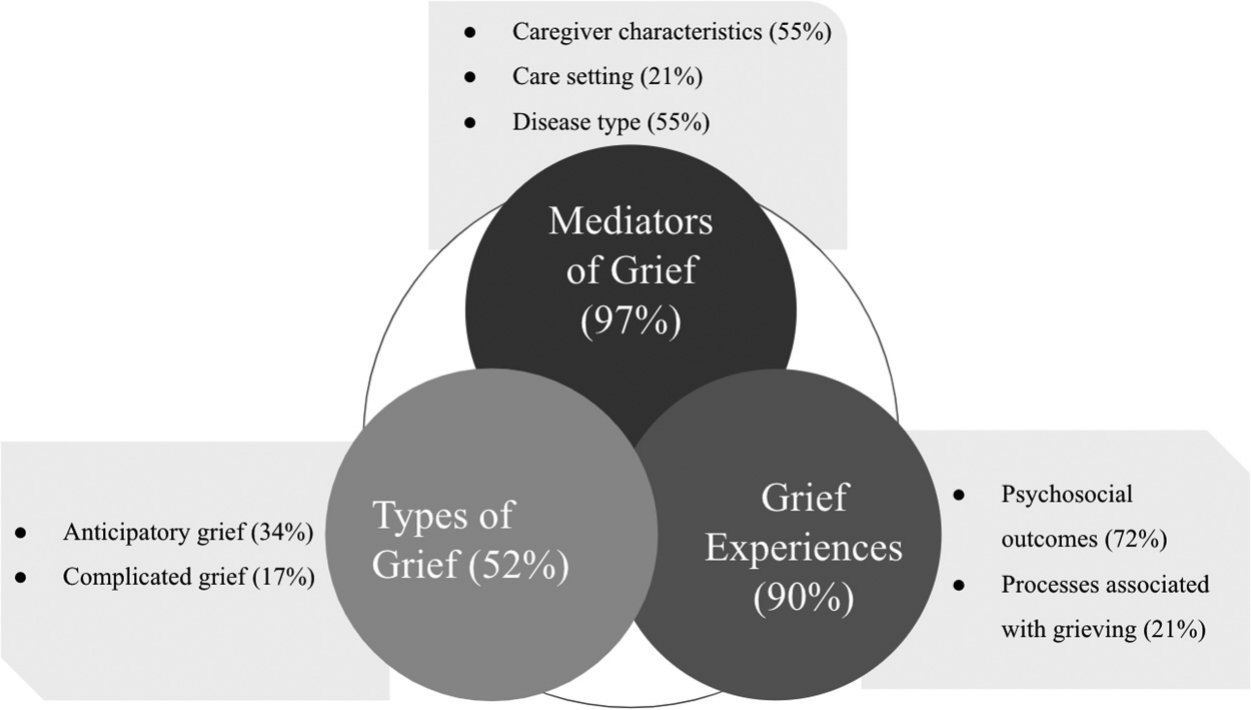
Scoping review themes and subthemes.

Initial selection results from electronic databases, advanced Google searches, and the Government of Canada website yielded 31 720 hits (Figure 1). Following deduplication, the titles and abstracts of the remaining studies were reviewed against the eligibility criteria. During the initial selection stage, 24 reviews that met the criteria were mined for references resulting in 18 relevant articles. In total, 258 sources were chosen for the full-text review stage. Twenty-nine articles met the inclusion criteria.^9,22–49^ All articles were peer-reviewed, with 10/29 being qualitative studies (34%),^9,22–30^ 17/29 quantitative studies (59%),^31–47^ and 2/29 employing mixed methods approaches (7%).^48,49^ All articles were from a North American context, with 5/29 (17%) being from Canada^23,24,26,27,30^ and the rest 24/29 (83%) being from the United States.^9,22,25,28,29,31–49^ Three main themes emerged from the thematic content analysis:^20^ (1) mediators of grief, (2) grief experiences, and (3) types of grief (Figure 2).

### Mediators of Grief

Ninety-seven percent of articles (28/29) explored factors which mediated the grief and bereavement experiences of ICs in North America.^9,22–46,48,49^ Factors were further divided into caregiver characteristics, care setting, and disease type of the care recipient.

#### Caregiver Characteristics

In total, 16/29 articles (55%) identified caregiver characteristics which influenced grief and bereavement experiences.^22,23,25,31–41,48,49^

##### Socioeconomic Status

Seven percent (2/29) of articles identified socioeconomic factors as contributing to heightened grief among ICs.^22,36^ In one study, difficulties paying for necessities was significantly associated with more depressive symptoms.^22^ Being employed was also associated with worsened complicated and general grief, suggesting that employment, and the stressors associated therein, can contribute to an even more difficult period of bereavement.^22,36^ Education was also shown to have an influence on grief, with low caregiver education associated with worsened grief.^22^

##### Demographics

Ten percent (3/29) of articles identified demographic factors, such as culture, race, and gender as influencing grief and bereavement.^36,41,49^ One article reported gender as a mediator of grief with most bereaved caregivers identifying as women.^36^ All 3 articles also examined the impact of culture and race/ethnicity on bereavement experiences.^36,41,49^ Differences in perceptions and expectations in regard to the role of the caregiver between cultures can influence individuals’ grief and bereavement experiences.^36,41,49^ For example, one study indicated that while coping strategies were similar among racial groups, European Americans had higher levels of perceived stress.^25^ The researchers suggested that the other racial groups in the study downplayed their stress as they either: (1) saw caregiving as part of their cultural duty, or (2) were not comfortable displaying those emotions.^25^ Cultural nuances such as this resulted in conclusions across multiple studies that cultural practices influenced end-of-life experiences.^36,41,49^

##### Caregiver Health Condition and Burden

Twenty-four percent (7/29) of articles reported caregiver health and burden as factors increasing caregiver grief.^22,34–36,38–40^ In one study, a higher number of chronic conditions in caregivers was associated with more depressive symptoms.^22^ A positive correlation between caregiver burden and heightened grief was found, including anticipatory and complicated grief.^35,36,38,39^

##### Time Spent Caregiving

One article (3%) reported that more time spent caregiving was associated with worsened grief.^22^ Those who dedicated more time to caregiving were found to have worsened depressive symptoms and complicated grief.^22^

##### Relationship to Patient

Twenty-eight percent (8/29) of articles examined caregiver/patient relationships in regard to grief.^23,31–33,35,37,41,48^ Adult children of the patient experienced a stronger form of grief characterized by guilt and frustration, while the spouse's grief was found to be quiet and sad.^48^ Another study reported an association between spousal status and a higher frequency of depressive symptoms.^32^ However, 3 studies reported no correlation between relationship to patient and grief/bereavement.^23,31,33^

#### Care Setting

In total, 6/29 articles (21%) identified the care setting as a mediator of grief and bereavement of ICs.^9,26,30,35,42,45^

##### Home-Based

Twenty-one percent (6/29) of articles discussed the impact that home-based hospice and palliative care had on the bereavement of ICs.^9,26,30,35,42,45^ Home-based caregiving was shown to result in positive and negative experiences for caregivers, with positive experiences outweighing negative ones.^30^ Positive experiences included a feeling of self-accomplishment and personal growth, while negative experiences included aversion to the place and feeling of failing to reduce suffering.^30^ Another study identified how a lack of support services for home-based caregivers could hinder the coping process, but that both formal and informal support could help.^26^

##### Hospice

Seven percent (2/29) of articles examined the hospice setting as a factor for caregiver grief.^42,45^ In one study, ICs of patients enrolled in a hospice 3 or fewer days before care receiver death were more likely to have major depressive disorder (24%) at follow-up compared to those whose care receiver had enrolled earlier (9%).^29^

#### Disease Type

In total, 16/29 articles (55%) identified disease type as a factor influencing the grief and bereavement of ICs.^23,24,26–30,32,36,38,39,42–44,46,48^

##### Cognitive Impairments: Dementia and Alzheimer's Disease

Thirty-one percent (9/29) of articles examined how ICs of dementia patients experience grief.^28,29,32,36,38,39,42,44,48^ One study reported greater grief and depression in dementia ICs compared to cardiac ICs.^44^ A common theme was relief, and in one study, many caregivers (72%) viewed the death of the care recipient as a relief to themselves—likely due to the complicated progression of dementia.^42^ Interestingly, caregivers who experienced relief had less complicated grief, and engaged in more leisure activities post bereavement.^32^ Caregivers also shared how grief changed as dementia progressed.^48^

##### Cancer

Twenty-four percent (7/29) of articles reported on the grief experiences of cancer ICs.^23,24,26,27,30,38,43^ The 2 themes primarily outlined were balance and hope. In one study, ICs viewed finding balance as “walking a fine line” between “deep grieving” and “moving forward”, with spirituality reported as a source of guidance.^27^ It was therefore unsurprising that another study found a main concern of ICs to be losing hope, which they combated through finding balance.^23^ Caregivers also spoke of the importance of finding strength from within, which helped with their coping process.^26^

### 2. Grief Experiences

In total, 26/29 articles (90%) reported on the common experiences that accompanied grief in ICs of hospice and palliative care patients.^9,22–24,26–37,39–48^ This included the psychosocial outcomes and the emotional processes that frequently occur during grieving.

#### Psychosocial Outcomes

Seventy-two percent (21/29) of articles reported on the psychosocial outcomes that could occur in tandem with grief.^22,23,28–37,39–47^ Sixty-nine percent (20/29) described negative psychosocial outcomes—including anxiety, despair, depression, distress, hopelessness, and hostility.^22,28–37,39–47^ Fifty-two percent (15/29) specifically reported on the experience of depression or depressive symptoms during the grieving period.^22,31,32,34,36–40,42–47^ The risk of depression in grieving caregivers was shown to correlate with caregiver rumination, higher perceived emotional suffering of patients, diminished feelings of relief, later enrollment in hospice and palliative care, patient's functional disability, unresolved regrets, and personal burden.^22,32,39,45,47^ Two articles (7%) noted that although grief might trigger depression/depressive symptoms, the 2 should be considered distinct emotional processes.^39,46^

Thirty-four percent (10/29) of articles reported positive psychosocial outcomes that were associated with grief in caregivers of palliative patients. This included relief, a sense of accomplishment, personal growth, a strengthened relationship, hope, and gratefulness.^22,23,28–30,32,35,37,42,46^ Experiences of relief were particularly common in the literature, with 24% (7/29) of articles reporting this emotion in grieving caregivers.^22,28,29,32,35,37,42^

Many articles acknowledged the competing and seemingly contradicting emotions and psychosocial outcomes that present themselves in grieving caregivers, with 27% (8/29) reporting both positive and negative aspects of the grieving experience.^22,28,29,32,35,37,42,45^

#### Processes Associated With Grieving

Twenty-one percent (6/29) of articles reported processes that grieving individuals commonly experienced.^9,23,24,26,27,36^ Seven percent of articles (2/29) focused on the stages of grief that caregivers experienced.^9,36^ One of these focused on the processes associated with anticipatory grief,^9^ and the other described trajectories of grief postdeath.^36^ Fourteen percent (4/29) focused on the processes of moving forward.^23,24,26,27^ Each of these articles spoke to the common experiences that grieving caregivers described as finding balance and moving on in their lives following the death of the patient.

### 3. Types of Grief

Fifty-two percent of articles (15/29) explored the types of grief that ICs experienced both before and after the death of a family member/friend.^9,22,24,25,28,29,32,34–36,38,43,44,48,49^ The 2 types of grief explored included anticipatory grief and complicated grief.

#### Anticipatory Grief

Thirty-four percent (10/29) of articles described the anticipatory grief experiences of ICs.^9,24,25,28,29,36,38,44,48,49^ This type of grief was experienced before the death of the care recipient and could entail the recognition of what is about to be lost, preparing for a life without the dying person, and experiencing feelings of freedom, but also isolation.^24,25,38^ One article explained that anticipatory grief was a gradual process made up of 5 different stages: realization, caretaking, presence, finding meaning, and transitioning.^9^ The process of caretaking, and increasing caregiver burden, might impede a caregiver's ability to experience anticipatory grief.^9,24,38^ One study found that anticipatory grief could follow multiple different trajectories.^36^ This study identified gender as a factor that determined an individual grief trajectory. Other studies identified found that factors such as ethnic and cultural background influenced a caregiver's anticipatory grief experience.^25,36^ This specific anticipatory grief experienced by the caregiver was associated with the care recipient losing the ability to eat ethnic foods, and participate in cultural events.^25,36^ Multiple studies referred to the benefits of anticipatory grief;^9,24,36^ however, one study indicated that anticipatory grief only resulted in temporary short-term relief immediately after death.^48^ Seven out of the 10 articles explored how anticipatory grief was exacerbated when caring for those with cognitive impairment diseases such as Alzheimer's and dementia.^28,29,36,38,44,48,49^ These studies consistently referred to the IC's loss of closeness with the dying person as a factor of their anticipatory grief. Two articles suggested that the anticipatory grief of dementia caregivers might be more complex than that of the general population, as they had to deal with the loss of the person as the disease progressed and memory deteriorated.^29,44^

#### Complicated Grief

Five out of 29 (17%) articles discussed the experience of complicated grief of ICs.^22,32,34,35,43^ Complicated grief or prolonged grief is grief that extends over a long period of time and is characterized by an inability to accept the death, preoccupation with thoughts of the deceased, and feelings of yearning/longing for the deceased.^34,43^ Factors that were found to be positively correlated with complicated grief in caregivers included the cognitive impairment of the dying person, caregiver burden, and being a spouse to the dying person.^22,34,43^ One study found that 20% of dementia caregivers experienced complicated grief.^35^ Additionally, high levels of prebereavement stress were correlated with prolonged grief experiences.^35^ One protective factor that was identified against complicated grief was the feeling of relief.^32^

## Discussion

Based on the emerging themes and findings from the 29 included sources,^9,22–49^ this section will present related practice, policy, and research implications. The common themes of grief and bereavement explored were: mediators of grief, grief experiences, and types of grief. Reoccurrences of these themes in the literature revealed the prevailing issues of grief and bereavement in ICs that need to be addressed.

### Practice Implications

There are several practice implications from these sources. In the literature, there is a call for more acknowledgment of the complexities of grief and bereavement by health care providers.^31,35,45^ Health care providers, especially those involved in bereavement interventions, should be offered education and training on the differences in roles and grief experiences of family members, especially between the role of caregiving assumed by the spouse and adult children.^33^ A great example of a recent campaign in acknowledging bereavement is the observation of “National Bereavement Day” in Canada. For the past 2 years, the CHPCA has hosted National Bereavement Day during the month of November to raise awareness of available bereavement resources for Canadians.^50^ The event includes programs that involve a diverse array of people from various health care fields and academia, from speakers in the “National Bereavement Day Webinar” to musicians paying tribute in “The Saying Goodbye Concert”.^50^

Those involved in bereavement services, such as nurses, social workers, and other health care professionals, must not only provide physical, psychosocial, spiritual care, and pain and symptom management, but also help prepare caregivers experiencing bereavement during end-of-life discussions and ongoing bereavement interventions.^28^ These bereavement interventions must also be designed with the mediators of grief in mind. For instance, the differences in experiences caused by chronic health conditions, cultural practices, and/or the care setting might influence the stage at which bereavement support and type of support offered would differ in efficacy.

After the loss of the care recipient, it is essential that resources and outlets for expression be shared with informal caregivers. An example of one such outlet is Death Cafes, where participants are encouraged to speak openly about their experiences with death.^51^ Providing these spaces and empowering caregivers to use them, can help them to process their grief during the bereavement period.^51^

### Policy Implications

At the policy level, there clearly is a lack of sufficient grief and bereavement support for caregivers. Caregivers need more resources and support to avoid potential psychological complications.^22^ Specifically, new policies should aim to have more resources for home-based care to help better prepare caregivers for the grief and bereavement period so that they may better cope during those challenging times.^9,42,49^ Recently, an Act to amend Bill C-220 (Bereavement Leave) passed through Canadian legislation.^52^ This amendment addresses previous gaps in the grief and bereavement space, by extending bereavement leave an extra 5 days.^52,53^ While this addresses a significant gap and provides individuals with increased time and space needed to grieve, future efforts should aim to increase uptake of public health interventions and address potential barriers to participation such as socioeconomic status and cultural perceptions.^22,36,41,49^

### Research Implications

There are a variety of research implications that stem from the results of this scoping review. For example, despite findings that a caregiver's culture/ethnicity can impact their grief and bereavement experience, only a limited subset of articles studied culture as a variable.^24,25,49^

Furthermore, one of the studies that did observe potential influences of culture on caregivers’ grief and bereavement experiences could not say so conclusively as participants were predominantly White of European descent.^25^ This suggests a need for increased diversity of participants in grief and bereavement research. Cultural and ethnic minorities must be represented more in studies, and it must be seen if and how cultural differences impact grief and bereavement.

There is also a need for further research into the impact of other caregiver characteristics, such as socioeconomic status, burden, health condition, and gender on the grief and bereavement experience. More specifically, further qualitative studies exploring why certain characteristics make individuals more likely to experience worsened grief would be an invaluable addition to the literature. For example, understanding why those that who employed experience heightened grief would allow for the creation of interventions that can address their unique stressors, including compassionate companies initiatives.^54,55^

Another mediator of grief that emerged from the literature was care setting. Research on the effects of the hospice care setting on the caregiver grief and bereavement experience is slightly less substantive when compared to the amount of research on home-based caregiving experiences. In addition to understanding the impact of hospice care setting, further research on specific factors which can affect the experience, such as time of enrollment, would be highly impactful.

The scoping review also observed that the dominant diseases of the care recipients in the 29 studies were cancer and cognitive impairment. While these 2 disease types have different life trajectories, it would be beneficial to explore other chronic, life-limiting, and terminal illnesses, especially in older adults. In a recent study on ICs of individuals with motor neurone disease, due to the uncertainty of imminent death brought on by the disease, caregivers experienced anticipatory grief and prolonged grief, with little acknowledgment by health care professionals and interventions to support them.^56^

Positive and negative psychosocial outcomes of grief experiences were also explored with a strong focus on depression/depressive symptoms. Depressive symptoms were detected prior to grief and assisted in increasing grief levels, usually first appearing during caregiving/predeath,^22,34,38^ as well as continuing or appearing during postbereavement.^31,36,37,43^ Other negative outcomes were often associated in studies exploring these depressive symptoms, such as distress and despair, and followed a similar pattern in increasing grief levels in caregivers.^33,34,36,43^

While both complicated grief and anticipatory grief appeared in the search, most articles exploring types of grief focused on anticipatory grief. These articles observed that lack of preparation for the care recipient's death and higher prebereavement distress due to lack of support resulted in difficulty transitioning out of mourning, and consequently prolonged and heightened bereavement.^9,24,43^ Further research on other types of grief such as delayed, disenfranchized, and cumulative grief would be a welcome addition to the North American literature.

### International Perspectives on Grief and Bereavement

Although this scoping review focused on the North American context, grief and bereavement literature spans the globe. Many of the gaps and areas of improvement mentioned above may have been addressed in other contexts. This section aims to shed light on some of the work that has already being done internationally to further grief and bereavement research.

An Australian study on the caregiver bereavement of family members with fatal motor neuron disease (MND) also focused on the psychosocial outcomes of bereavement.^57^ While not as prevalent in the North American literature, the fatality of MND resulted in the experience of prolonged grief in caregivers which, similar to our review, saw an association between prolonged grief and the negative psychosocial outcomes of depression and anxiety.^22,34,57^ Types of grief and the grief experiences associated with them were similar to the international literature.

The link between grief and psychosocial outcomes was further explored in another study in Portugal. In this study, the authors aimed to conceptualize anticipatory grief in ICs of cancer patients.^58^ Interestingly, the categories the study used for grief experiences were: *traumatic distress, separation distress,* and *emotional regulation and dysregulation*.^58^ This categorization of themes or subthemes in IC bereavement research might be beneficial as it offers a different lens in viewing grief experiences—going beyond the mainstream grouping of grief experiences as negative and positive psychosocial outcomes.

### Grief During COVID-19

The COVID-19 pandemic has greatly impacted the manner in which individuals are experiencing hospice and palliative/end-of-life care, grief, and bereavement.^59–61^ Changes resultant of the pandemic have impacted emotional and financial aspects of caregivers’ lives.^60^ These changes, such as the inability to visit a dying person, loss of financial security, and modified end-of-life practices, have implications for the way that grief manifests.^60^ There has also been a loss in lifestyle, cultural, and social practices due to the pandemic.^59^ Although research has not yet addressed the psychological impact of COVID-19 on informal caregivers, it can be reasonably assumed that this pandemic, and the unique circumstances which accompany it, can exacerbate feelings of depression, anxiety, and loneliness.^60^

It is important for caregivers to stay connected with their social supports and for policy and decision-makers to prepare for a potential increase in the need for mental health and grief support following the pandemic.^61^ Spiritual care resources and the increased uptake of virtual grief supports/resources were instrumental in addressing both caregiver and staff needs. Given the losses endured in the pandemic, there is a pressing need for these supports to be enhanced and sustained in the coming month.^62^

In addition, the establishment of a COVID-19 task force that is specially set to address the aforementioned issues and gaps via stakeholder consultations. These unique circumstances can also prompt a showcase highlighting the importance of personal goodbyes and the distinct grief and bereavement being faced by informal caregivers at this time.^63,64^ It is also imperative that organizations within the hospice palliative care space, such as Pallium Canada and Canadian Virtual Hospice, continue to share lessons learned and tools created in response to the pandemic addressing the unique needs of informal caregivers.^62^

### Strengths and Limitations

Strengths of our review include a broad exploration of the grief and bereavement experiences of ICs in the North American hospice and palliative care realm. Furthermore, a comprehensive and systematic approach in line with Arksey and O’Malley's framework ensured that the broadest possible viewpoints were obtained and addressed.^19^

The limitations of our study are several. It might be hypothesized that the inclusion of only English literature while exploring the bereavement experience in a multilingual North American context might provide limited perspective. In addition, quality assessment of studies included was precluded in an effort to obtain a broad view of the landscape. The reasons for the former were twofold, including lack of ready access to translators as well as a paucity of research from Canada. The inclusion of studies from Canada and the United States could be considered both a strength and limitation. The health care systems of the 2 countries are different; therefore availability, access, and financial constraints, etc manifest differently. However, the common core values of the caregivers provide a commonality to understand the challenges faced by this often-overlooked set of caregivers. Finally, no reviewed gray literature was included in the final scoping review stage, as none did meet the inclusion criteria. One reason for this limitation is that the majority of gray literature focused on interventions for grief and bereavement as opposed to the experiences of informal caregivers. As a result, all studies in this scoping review are peer-reviewed. Despite this, the research methods vary from cohort studies, secondary analyses of randomized control studies to prospective studies.

## Conclusions

As more individuals face the reality of informal caregiving, there is a pressing need to understand their grief and bereavement experiences. The factors identified provide valuable insight when developing support. In addition, the COVID-19 pandemic has highlighted further issues, causing grief to become “suspended” when family and friends are prevented from mourning together.^65^ The competing emotions and psychosocial outcomes experienced by caregivers reflect the nuanced nature of their care. Understanding the nuances of informal caregivers’ experiences with grief and bereavement will inform and advance practice, policy, and research. Practitioners should be further educated on how to properly acknowledge the complexity of grief and bereavement for ICs, specifically paying attention to mediators of grief and culture.

## Appendix (Supplemental Material—Online Only)

**Table:** AgeLine search string

Search number	Query
1	palliative care OR terminal care OR hospice care
2	hospices
3	palliative medicine
4	terminally ill
5	(terminal* OR hospice OR end-of-life OR palliative OR supportive OR comfort) n4 care)
6	(end stage* n5 [disease* OR ill* OR care or caring])
7	([incurable OR advanced] n5 [ill* OR disease*])
8	EoLC
9	(palliative n4 [treat* OR medicine])
10	1 OR 2 OR 3 OR 4 OR 5 OR 6 OR 7 OR 8 OR 9
11	bereavement OR grief OR disenfranchized grief
12	emotional regulation
13	psychological distress
14	sadness
15	psychology
16	bereav*
17	loss*
18	grief
19	griev*
20	mourn*
21	psychological adaptation
22	11 OR 12 OR 13 OR 14 OR 15 OR 16 OR 17 OR 18 OR 19 OR 20 OR 21
23	caregivers
24	family relations
25	adult children
26	siblings OR spouses
27	family
28	friends
29	caregiv*
30	([famil* OR friend* OR relative* OR spous* OR partner* OR husband* OR wife OR son* OR daughter* OR child* OR sibling* OR brother* OR sister* OR informal] n5 [care* OR care giv*])
31	23 OR 24 OR 25 OR 26 OR 27 OR 28 OR 29 OR 30
32	10 AND 22 AND 31

**Table:** CINAHL search string

Search number	Query
1	palliative care OR terminal care OR hospice care
2	hospices
3	palliative medicine
4	terminally ill
5	([terminal* OR hospice OR end-of-life OR palliative OR supportive OR comfort] n4 care)
6	(end stage* n5 [disease* OR ill* OR case OR caring])
7	“([incurable OR advanced] n5 [ill* OR disease*])”
8	([incurable OR advanced] n5 [ill* OR disease*])
9	EoLC
10	(palliative n4 [treat* OR medicine])
11	1 OR 2 OR 3 OR 4 OR 5 OR 6 OR 7 OR 8 OR 9 OR 10
12	bereavement OR grief OR disenfranchized grief
13	emotional regulation
14	psychological distress
15	sadness
16	psychology.fs
17	psychology
18	bereav*
19	loss*
20	grief
21	griev*
22	mourn*
23	(MH “Adaptation, Psychological + ”)
24	12 OR 13 OR 14 OR 15 OR 17 OR 18 OR 19 OR 20 OR 21 OR 22 OR 23
25	caregivers
26	family relations
27	adult children
28	siblings or spouses
29	family
30	friends
31	caregiv*
32	([famil* OR friend* OR relative* OR spous* OR partner* OR husband* OR wife OR son* OR daughter* OR child* OR sibling* OR brother* OR sister* OR informal] n5 [care* OR caregiv*])
33	25 OR 26 OR 27 OR 28 OR 29 OR 30 OR 31 OR 32
34	11 AND 24 AND 33

**Table:** Cochrane Library search string

Search number	Query
1	(mh ^“palliative care”) OR (mh ^“hospice care”) OR (mh ^“terminal care”)
2	(mh ^Hospices)
3	(mh ^“Hospice and Palliative Care Nursing”)
4	(mh ^“Palliative Medicine”)
5	(mh ^“Terminally Ill”)
6	([terminal* OR hospice OR end-of-life OR palliative OR supportive OR comfort] near/4 care):ti,ab,kw
7	(end stage* near/5 [disease* OR illn* OR care OR caring]):ti,ab,kw
8	([incurable OR advanced OR terminal*] near/5 [illn* OR disease*]):ti,ab,kw
9	(palliati* OR EoLC OR hospice* OR terminal* OR end-of-life):ti,ab,kw
10	(palliative near/4 [treat* OR medicine OR therap*]):ti,ab,kw
11	{OR #1-#10}
12	(mh ^bereavement) OR (mh ^grief) OR (mh ^“disenfranchized grief”)
13	(mh ^“emotional regulation”)
14	(mh ^“psychological distress”)
15	(mh ^sadness)
16	psychology:ti,ab,kw
17	bereav*:ti,ab,kw
18	loss*:ti,ab,kw
19	grief:ti,ab,kw
20	griev*:ti,ab,kw
21	mourn*:ti,ab,kw
22	(mh ^“adaptation, Psychological”)
23	{OR 12 to 22}
24	(mh ^Caregivers)
25	(mh ^“Family Relations”)
26	(mh ^“Adult Children”)
27	(mh ^siblings) OR (mh ^spouses)
28	(mh ^Family)
29	(mh ^Friends)
30	caregiv*:ti,ab,kw
31	([famil* OR friend* OR relative* OR spous* OR partner* OR husband* OR wife OR son* OR daughter* OR child* OR sibling* OR brother* OR sister* OR informal] near/5 [care* OR care giv*]):ti,ab,kw
32	{OR #24-#31}
33	11 AND 23 AND 32

**Table:** EMBASE search string

Search number	Query
1	palliative care/
2	terminal care/ or hospice care/
3	hospice/
4	"Hospice and Palliative Care Nursing"/
5	palliative therapy/ OR cancer palliative therapy/
6	terminally ill patient/ OR hospice patient/
7	([terminal* OR hospice OR end-of-life OR palliative OR supportive OR comfort] adj4 care).tw,kw.
8	(end stage* adj5 [disease* OR illn* OR care OR caring]).tw,kw.
9	([incurable OR advanced OR terminal*] adj5 [illn* OR disease*]).tw,kw.
10	(palliati* OR hospice* OR terminal OR end-of-life OR EoLC).tw,kw.
11	(palliative adj4 [treat* OR medicine OR therap*]).tw,kw.
12	1 OR 2 OR 3 OR 4 OR 5 OR 6 OR 7 OR 8 OR 9 OR 10 OR 11
13	bereavement/ or bereavement counseling/ or bereavement support/
14	grief/ OR anticipatory grief/ OR disenfranchized grief/ OR mourning/ OR prolonged grief/ OR sorrow/ OR unresolved grief/
15	complicated grief/
16	emotion regulation/
17	distress syndrome/
18	sadness/
19	bereav*.tw,kw.
20	loss*.tw,kw.
21	grief.tw,kw.
22	griev*.tw,kw.
23	mourn*.tw,kw.
24	coping behavior/
25	coping behavior assesment/ OR coping health inventory for parents/ OR coping strategy questionnaire/
26	13 OR 14 OR 15 OR 16 OR 17 OR 18 OR 19 OR 20 OR 21 OR 22 OR 23 OR 24 OR 25
27	caregiver/
28	caregiver support/
29	family coping/
30	family relation/ OR family/
31	friend/
32	spouse/ OR domestic partner/ OR husband/ OR wife/
33	sibling/
34	brother/ OR sister/
35	caregiv*.tw,kw.
36	([famil* OR friend* OR relative* OR spous* OR partner* OR husband* OR wife OR son* OR daughter* OR child* OR sibling* OR brother* OR sister* OR informal] adj5 [care* OR care giv*]).tw,kw.
37	27 OR 28 OR 29 OR 30 OR 31 OR 32 OR 33 OR 34 OR 35 OR 36
38	12 AND 26 AND 37

**Table:** JSTOR search string

Search number	Query
1	palliative
2	"terminal care"
3	hospice care
4	end of life
5	1 OR 2 OR 3 OR 4
6	bereav*
7	grief
8	loss
9	"emotional adjustment"
10	6 OR 7 OR 8 OR 9
11	caregivers
12	famil* NEAR 4 caregiv*)
13	famil* NEAR 4 relat*
14	11 OR 12 OR 13
15	5 AND 10 AND 14

**Table:** MEDLINE search string

Search number	Query
1	palliative care/ OR terminal care/ OR hospice care
2	Hospices/
3	"Hospice and Palliative Care Nursing"/
4	Palliative Medicine/
5	Terminally Ill/
6	([terminal* OR hospice OR end-of-life OR palliative OR supportive OR comfort] adj4 care).tw,kf.
7	(end stage* adj5 [disease* OR illn* OR care or caring]).tw,kf.
8	([incurable OR advanced or terminal*] adj5 [illn* OR disease*]).tw,kf.
9	(palliati* OR EoLC OR hospice* OR terminal* OR end-of-life).tw,kf.
10	(palliative adj4 [treat* OR medicine OR therap*]).tw,kf.
11	1 OR 2 OR 3 OR 4 OR 5 OR 6 OR 7 OR 8 OR 9 OR 10
12	bereavement/ or grief/ or disenfranchized grief/
13	Emotional Regulation/
14	psychological distress/
15	sadness/
16	bereav*.tw,kf.
17	loss*.tw,kf.
18	grief.tw,kf.
19	griev*.tw,kf.
20	mourn*.tw,kf.
21	adaptation, Psychological/
22	Caregivers/
23	Family Relations/
24	Adult Children/
25	siblings/ or spouses/
26	Family/
27	Friends/
28	caregiv*.tw,kf.
29	([famil* OR friend* OR relative* OR spous* OR partner* OR husband* OR wife OR son* OR daughter* OR child* OR sibling* OR brother* OR sister* OR informal] adj5 [care* OR care giv*]).tw,kf.
30	22 OR 23 OR 24 OR 25 OR 26 OR 27 OR 28 OR 29
31	12 OR 13 OR 14 OR 15 OR 16 OR 17 OR 18 OR 19 OR 20 OR 21
32	11 AND 30 AND 31

**Table:** Project Muse search string

Search number	Query
1	“palliative care/”
2	“hospice care/”
3	hospice/
4	1 OR 2 OR 3
5	bereavement/
6	grief/
7	mourning/
8	“anticipatory grief/”
9	sadness/
10	5 OR 6 OR 7 OR 8 OR 9
11	caregiver/
12	family/
13	husband/
14	wife/
15	brother/
16	sister/
17	11 OR 12 OR 13 OR 14 OR 15 OR 16
18	4 AND 10 AND 17

**Table:** ProQuest Sociological Abstracts search string

Search number	Query
1	su(“palliative care”) OR su(“terminal care”) OR su(“hospice care”)
2	su(“palliative medicine”) OR su(“palliative nursing”) OR su(“hospice nursing”)
3	su(“end-of-life care”) OR su(“end-of-life service”) OR su(“end-of-life treatment”)
4	su(“comfort care”) OR su(“supportive care”) OR su(“supportive treatment”) OR su(“supportive therapy”)
5	noft(“terminal care”) OR noft(“hospice care”) OR noft(“palliative care”)
6	noft(“end-of-life care”) OR noft(“palliative care”) OR noft(“palliating”)
7	noft(“comfort care”) OR noft(“advanced illness”) OR noft(“supportive treatment”)
8	noft(“terminal*”) OR noft(“palliat*”) OR noft(“dying”)
9	noft(“palliative treatment”) OR noft(“palliative medicine”) OR noft(“palliative therapy”)
10	mainsubject.Exact(“palliative care”) OR mainsubject.Exact(“hospice care” OR “hospices” OR “hospice/hospices” OR “palliative care”) OR mainsubject.Exact(“terminal diseases” OR “terminal illnesses” OR “terminally ill people” OR “hospice care” OR “terminally ill” OR “hospices” OR “terminal care” OR “hospice/hospices” OR “terminal illness” OR “palliative care”)
11	(end-stage* OR “end stage*”) NEAR/4 (disease* OR ill* OR care OR caring)
12	(palliat* OR hospice OR terminal) NEAR/4 (care* OR nurs* OR medicine OR treatment OR therapy OR comfort)
13	1 to 12
14	su(grie*) OR su(bereav*) OR su(loss)
15	su(coping) OR su(adjust*) OR su(sad*)
16	su(mourn*) OR su(pastoral) OR su(suffer*)
17	su(mourn*) OR su(pastoral) OR su(suffer*)
18	su(“normal grief”) OR su(“distorted grief”) OR su(“absent grief”) OR su(“masked grief”)
19	noft(“normal grief”) OR noft(“distorted grief”) OR noft(“absent grief”) OR noft(“masked grief”)
20	noft(“anticipatory grief”) OR noft(“delayed grief”) OR noft(“complicated grief”)
21	su(“anticipatory grief”) OR su(“delayed grief”) OR su(“complicated grief”)
22	mainsubject.Exact(“disenfranchized grief” OR “bereavement” OR “palliative care” OR “anticipatory grief” OR “grief”) OR mainsubject.Exact(“disenfranchized grief” OR “palliative care” OR “anticipatory grief” OR “grief”) OR mainsubject.Exact(“disenfranchized grief” OR “mourning” OR “bereavement” OR “palliative care” OR “anticipatory grief” OR “grief”)
23	(grie* OR bereav* OR mourn) NEAR/4 (pyschol* OR adapt* OR adjust* OR comfort OR unresolved OR complex OR chronic)
24	(grie* OR bereav* OR mourn) NEAR/4 (pastoral* OR spirit* OR support* OR disenfranchized)
25	(grie* OR bereav* OR mourn) NEAR/4 (normal OR anticipatory OR complex OR masked OR distorted OR complicated OR delayed OR absent)
26	14 to 25
27	su(care*) OR su(caregiv*) OR su(“informal caregiver”)
28	noft(care*) OR noft(caregiv*) OR noft(“informal caregiver”)
29	noft(relati*) OR noft(family) OR noft(friends)
30	su(relati*) OR su(family) OR su(“support system”) OR su(friend*)
31	su(partner) OR su(spouse) OR su(common-law) OR su(husbands) OR su(wives)
32	noft(partner) OR noft(spouse) OR noft(common-law) OR noft(husbands) OR noft(wives)
33	noft(sibling) OR noft(child*) OR noft(“adult offspring”) OR noft(sister) OR noft(brother)
34	mainsubject.Exact(“spouses” OR “spouse/spousal” OR “adaptation, psychological; social support; sociology, medical; stress, psychological”) OR mainsubject.Exact(“spouses” OR “adult-child interactions” OR “spouse/spousal” OR “adult child relationship” OR “adult children” OR “adaptation, psychological; social support; sociology, medical; stress, psychological” OR “child”) OR mainsubject.Exact(“spouses” OR “adult-child interactions” OR “spouse/spousal” OR “adult child relationship” OR “adult children” OR “dependent/dependents” OR “dependents” OR “adaptation, psychological; social support; sociology, medical; stress, psychological” OR “child”) OR mainsubject.Exact(“spouses” OR “siblings” OR “adult children” OR “dependent/dependents” OR “adaptation, psychological; social support; sociology, medical; stress, psychological” OR “adult-child interactions” OR “spouse/spousal” OR “adult child relationship” OR “dependents” OR “sibling relations” OR “sibling/siblings” OR “sibling relationships” OR “child”) OR mainsubject.Exact(“friendship” OR “close friends” OR “close friendships” OR “friend/friends/friendship” OR “friendships” OR “friends”) OR mainsubject.Exact(“support networks” OR “friendship” OR “support systems” OR “close friends” OR “close friendships” OR “friend/friends/friendship” OR “supports” OR “friendships” OR “support” OR “friends” OR “supported living” OR “adaptation, psychological; social support; sociology, medical; stress, psychological”)
35	(care* OR caregiv* OR “informal caregiver”) NEAR/4 (famil* OR relat* OR support* OR sibling OR spouse OR wife OR husband OR partner OR child* OR son OR daughter) .
36	(care* OR caregiv* OR “tak* care”) NEAR/4 (family OR relative OR support*)
37	27 to 36
38	13 AND 26 AND 37

**Table:** PsycINFO search string

Search number	Query
1	palliative care/ OR “death and dying”/ OR hospice/ OR terminally ill patients/
2	([terminal* OR hospice OR end-of-life OR palliative OR supportive OR comfort] adj4 care).mp.
3	(end stage* adj5 [disease* OR illn* OR care OR caring]).mp.
4	([incurable OR advanced or terminal*] adj5 [illn* OR disease*]).mp.
5	(palliati* OR EoLC OR hospice* OR terminal* OR end-of-life).mp.
6	(palliative adj4 [treat* OR medicine OR therap*]).mp.
7	(hospice* OR terminal* OR end-of-life).mp
8	1 OR 2 OR 3 OR 4 OR 5 OR 6 OR 7
9	bereavement/ or grief/
10	grief counseling/ OR counseling/ OR life experiences/ OR traumatic loss/
11	emotional regulation/
12	coping behavior/ OR emotional adjustment/ OR “stress and coping measures”/
13	sadness/
14	bereav*.mp.
15	loss*.mp.
16	grief.mp.
17	griev*.mp.
18	mourn*.mp.
19	9 OR 10 OR 11 OR 12 OR 13 OR 14 OR 15 OR 16 OR 17 OR 18
20	caregivers/
21	family relations/ OR family/ OR sibling relations/
22	spouses/ OR family members/ OR husbands/ OR wives/
23	adult offspring/
24	caregiv*.mp.
25	([famil* OR friend* OR relative* OR spous* OR partner* OR husband* OR wife OR son* OR daughter* OR child* OR sibling* OR brother* OR sister* OR informal] adj5 [care* OR care giv*]).mp.
26	20 or 21 or 22 or 23 or 24 or 25
27	8 AND 19 AND 26

**Table:** Scopus search string

Search number	Query
1	“palliative care/”
2	“terminal care/” OR “hospice care/”
3	hospice/
4	“palliative therapy/” OR “cancer palliative therapy/”
5	“terminally ill patient/” OR “hospice patient/”
6	([terminal* OR hospice OR “end-of-life” OR palliative OR supportive OR comfort] w/4 care)
7	(“end stage”* w/5 [disease* OR illn* OR care OR caring])
8	(palliati* OR hospice* OR terminal OR “end-of-life” OR eolc)
9	(palliative w/4 [treat* OR medicine OR therap*])
10	1 OR 2 OR 3 OR 4 OR 5 OR 6 OR 7 OR 8 OR 9 OR 10
11	bereavement/ OR “bereavement counseling/” OR “bereavement support/”
12	grief/ OR “anticipatory grief/” OR “disenfranchized grief/” OR mourning/ OR “prolonged grief/” OR sorrow/ OR “unresolved grief/”
13	“complicated grief/”
14	sadness/
15	bereav*
16	loss*
17	Grief
18	griev*
19	mourn*
20	“coping behavior/”
21	11 OR 12 OR 13 OR 14 OR 15 OR 16 OR 17 OR 18 OR 19 OR 20
22	caregiver/
23	“family coping/”
24	“family relation/” OR family/
25	spouse/ OR “domestic partner/” OR husband/ OR wife/
26	sibling/
27	brother/ OR sister/ OR cousin/
28	caregiv*
29	([famil* OR friend* OR relative* OR spous* OR partner* OR husband* OR wife* OR son* OR daughter* OR child* OR sibling* OR brother* OR sister* OR informal] w/5 care* OR care giv*)
30	22 OR 23 OR 24 OR 25 OR 26 OR 27 OR 28 OR 29
31	10 AND 21 AND 30
